# Early Detection of Public Health Emergencies of International Concern through Undiagnosed Disease Reports in ProMED-Mail

**DOI:** 10.3201/eid2602.191043

**Published:** 2020-02

**Authors:** Camille Rolland, Clément Lazarus, Coralie Giese, Bastien Monate, Anne-Sophie Travert, Jérôme Salomon

**Affiliations:** Public Health Emergency Operations Center of the French Ministry of Health, Paris, France (C. Rolland, C. Lazarus, B. Monate, A.-S. Travert);; Ministry of Health, Paris (C. Giese, J. Salomon)

**Keywords:** surveillance, outbreaks, emerging infectious diseases, global health, World Health Organization, ProMED-mail, early detection, public health emergencies of international concern, undiagnosed diseases, international health regulations, health security, Disease Outbreak News

## Abstract

We conducted a retrospective analysis of all reports in ProMED-mail that were initially classified as undiagnosed diseases during 2007–2018. We identified 371 cases reported in ProMED-mail; 34% were later diagnosed. ProMED-mail could be used to supplement other undiagnosed disease surveillance systems worldwide.

To preserve human health security, a global surveillance system able to rapidly detect, verify, and assess burgeoning outbreaks is key. The World Health Organization (WHO) International Health Regulations (2005) (*1*) provides an international and legally binding framework for the early detection of, reporting of, and response to any public health threat (e.g., infectious disease outbreaks) that might be of international concern using an all-hazards approach (*2*).

Event-based surveillance through informal sources now represents a critical source for epidemic intelligence (*3*). Almost all major outbreaks during 1994–2017 investigated by the WHO were early reported and identified through informal sources (*4*–*7*). One of the most valued, internationally acknowledged sources for epidemic intelligence activities that is also available as an open source is ProMED-mail (*4*,*8*). By relying on local media, professional networks, and on-the-ground experts, ProMED-mail staff produce reports on occurrences of emerging infectious diseases and outbreaks in near real-time. Specialist moderators curate these reports and provide subject matter expert commentaries.

ProMED-mail captures many reports of undiagnosed diseases (i.e., reports of public health events for which the diagnosis has not yet been found or reported by field professionals and cannot be classified). Events in these reports take place all around the world, and reports are provided without enough information to formulate a comprehensive risk assessment.

Even though an undiagnosed disease report in ProMED-mail might be an early signal of a major future event (e.g., outbreak), such reports have not been described in the literature. In this study, we aimed to provide a descriptive analysis of reports of undiagnosed disease events related to human health published on ProMED-mail since 2007 to determine whether these reports should be considered in further risk assessments.

## The Study

**Figure 1:**
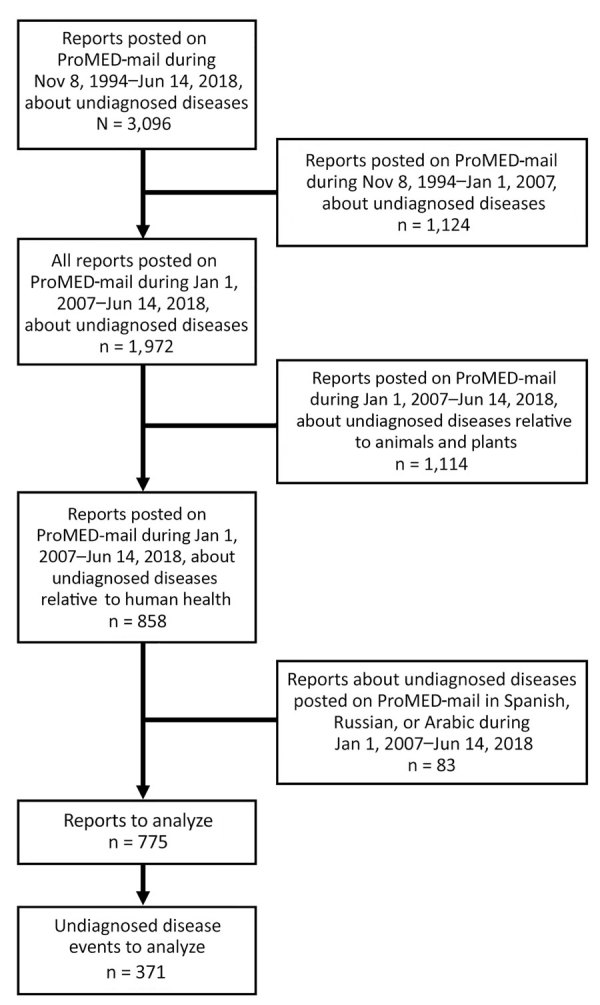
Selection of ProMED-mail reports to analyze for undiagnosed disease events related to human health, January 1, 2007–December 30, 2017.

We conducted a retrospective analysis of all reports of undiagnosed diseases in the ProMED-mail registry that were published during January 1, 2007–June 14, 2018 (Figure 1). ProMED-mail staff provided all the archives for undiagnosed diseases and unknown diseases relative to humans, animals, and plants. We also collected data directly from Disease Outbreak News on the WHO website (https://www.who.int/csr/don/archive/country) for the period of the study; these data are also open access and disseminated as specified by Article 11 of the International Health Regulations (2005). From ProMED-mail reports, we collected data on case location (WHO zone), source of information, date of publication, number of cases, geographic distribution (i.e., regional, national, or international) of cases, affected population, and final diagnosis. We searched the WHO website for the existence of a related Disease Outbreak News report. We sought information on the confirmation (i.e., biologic confirmation) of the final diagnosis both in subsequent ProMED-mail reports and in Disease Outbreak News reports. When several notifications were linked to 1 undiagnosed disease event, we made the link between notifications using the date of occurrence and location data. We described quantitative variables using median and range and qualitative variables using percentages.

**Figure 2:**
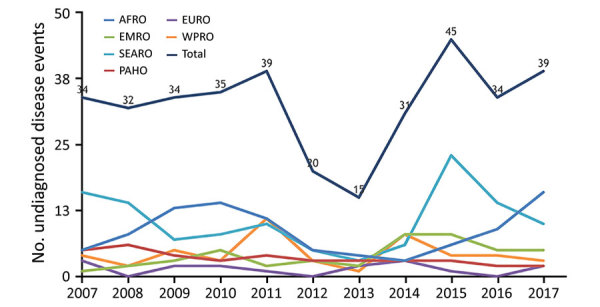
Undiagnosed disease events in humans posted on ProMED-mail, by location (World Health Organization zone), January 1, 2007–December 30, 2017. AFRO, African Regional Office; EMRO, Eastern Mediterranean Regional Office; EURO, Europe Regional Office; PAHO, Pan American Health Organization; SEARO, South-East Asia Regional Office; WPRO, Western Pacific Regional Office.

During January 1, 2007–June 14, 2018, a total of 775 ProMED-mail reports accounted for 371 individual undiagnosed disease events in humans (Figure 1). The median number of undiagnosed disease events per year was 34 (range 15–45) (Figure 2). The sources of these reports were mainly the national press (67%, 250/371); 25% (93/371) were from international media, and 8% (28/371) were from experts.

The countries most affected by undiagnosed diseases were India (68/371), Sudan (20/371), Bangladesh (16/371), Nepal (15/371), China (14/371), the Democratic Republic of the Congo (13/371), Uganda (13/371), the United States (13/371), Vietnam (11/371), and Nigeria (9/371) (Appendix Figure). Overall, 44% of undiagnosed disease events were in rural areas and 56% were in urban areas; 8% (31/371) took place within a capital city. For 54% (200/371) of undiagnosed disease events, no specific population (e.g., children <18 years of age, persons >65 years of age, health professionals) was identified. For 2.4% (9/371) of undiagnosed disease events, healthcare professionals were affected.

We found a Disease Outbreak News report for 6.5% (24/371) of the undiagnosed disease events described in ProMED-mail. The median delay between the first ProMED-mail notification and the Disease Outbreak News publication was 18.5 (range –1 to 254) days (Table).

**Table:** Characteristics of the 24 undiagnosed disease events posted on ProMED-mail that were also published in Disease Outbreak News, 2007–2018*

ProMED-mail diagnosis date	Country	WHO zone	Diagnosis	Publication date	Period, d†
2007 Sep 1	DRC	AFRO	Ebola	2007 Aug 31	–1
2007 Nov 9	Angola	AFRO	Bromide poisoning	2007 Nov 16	7
2008 Mar 8	Senegal	AFRO	Lead poisoning	2008 Jun 28	112
2008 Jun 23	Senegal	AFRO	Lead poisoning	2008 Jun 28	5
2008 Aug 21	China	WPRO	Powder milk intoxication	2008 Sep 18	28
2008 Oct 5	South Africa	AFRO	Lassa fever	2008 Oct 10	5
2008 Oct 9	DRC	AFRO	Ebola	2008 Dec 26	78
2008 Dec 22	DRC	AFRO	Ebola	2008 Dec 26	4
2010 Sep 27	Pakistan	EMRO	Crimean-Congo hemorrhagic fever	2010 Oct 25	28
2011 Aug 16	Angola	AFRO	Undiagnosed disease	2011 Aug 15	–1
2012 Jul 4	Cambodia	WPRO	Hand, foot and mouth disease	2012 Jul 4	0
2012 Jul 25	Uganda	AFRO	Ebola	2012 Jul 27	2
2012 Aug 2	Uganda	AFRO	Ebola	2012 Aug 3	1
2012 Dec 23	Sudan	EMRO	Yellow fever	2012 Nov 13	–41
2014 Feb 24	USA	PAHO	Human enterovirus infection	2014 Sep 17	205
2015 Feb 9	Brazil	PAHO	Zika virus infection	2015 Oct 21	254
2015 Jul 20	Egypt	EMRO	Dengue fever	2015 Nov 12	115
2015 Aug 15	Egypt	EMRO	Dengue fever	2015 Nov 12	90
2015 Sep 5	Senegal	AFRO	Chikungunya	2015 Sep 14	9
2016 Jun 25	South Sudan	EMRO	Undiagnosed disease	2016 May 19	–38
2016 Jan 22	Angola	AFRO	Yellow fever	2016 Feb 12	21
2017 Mar 8	Nigeria	AFRO	Meningitis	2017 Mar 24	16
2017 Apr 26	Liberia	AFRO	Meningitis	2017 May 5	9
2018 Jan 7	Kenya	AFRO	Chikungunya	2018 Feb 27	51

*AFRO, African Regional Office; DRC, Democratic Republic of the Congo; EMRO, Eastern Mediterranean Regional Office; PAHO, Pan American Health Organization; WHO, World Health Organization; WPRO, Western Pacific Regional Office. †Time between notification on ProMED-mail and official publication in Disease Outbreak News (https://www.who.int/csr/don/archive/country). Negative numbers indicate publication in Disease Outbreak News first.

A final diagnosis was found for 34% (127/371) of undiagnosed disease events (Appendix Table). Among the 127 events for which a final diagnosis could be determined, the most frequent diseases were chikungunya (6/127), leptospirosis (6/127), Nipah virus infection (6/127), Ebola (5/127), meningitis (5/127), yellow fever (5/127), anthrax (4/127), Crimean-Congo hemorrhagic fever (4/127), and nodding disease (4/127).

Undiagnosed diseases might be reported in various medical situations. They often occur in cases of delayed diagnosis of common diseases when access to appropriate medical care or services (e.g., epidemiologic investigations, laboratory testing) is limited. As such, most undiagnosed disease events occurred within low-resource countries. Undiagnosed disease reports less frequently account for unusual or unexpected diseases, such as imported or emerging diseases.

Reporting of undiagnosed diseases through ProMED-mail can be limited by climatic or geopolitical events in the region, which was probably reflected by the yearly and geographic variation in the reporting of undiagnosed disease events we observed. Although informal sources of public health information are valuable, the editorial content of the news sources the reports are based on can strongly limit their usefulness. Hence, our data analysis was limited by missing data.

## Conclusions

The impact of ProMED-mail on the public health emergency preparedness response is reflected by the percentage of undiagnosed disease events published through this informal reporting system (6.5%) that were also shared internationally through WHO’s Disease Outbreak News website. Regions and countries could benefit from complementing their undiagnosed disease surveillance systems with ProMED-mail (*9*). Using this approach would help further establish undiagnosed disease event-based monitoring as an invaluable public health tool. ProMED-mail provides critical content and an alternative to standard indicator-based outbreak reporting for undiagnosed diseases (*4*).

Appendix More information about early detection of public health emergencies of international concern through undiagnosed disease reports in ProMED-mail.
