# Supplementary material for: Early Detection of Public Health Emergencies of International Concern through Undiagnosed Disease Reports in ProMED-Mail
Source: Emerg Infect Dis. 2020 Feb;26(2):336–9. doi: 10.3201/eid2602.191043 (PMC6986859; doi:10.3201/eid2602.191043)
Supplement: Appendix — More information about early detection of public health emergencies of international concern through undiagnosed disease reports in ProMED-mail. [file 19-1043-Techapp-s1.pdf]

Article DOI: https://doi.org/10.3201/eid2602.191043

Early Detection of Public Health Emergencies of International Concern through Undiagnosed Disease Reports in ProMED-Mail

Appendix

Appendix Table. Undiagnosed disease events in which diagnoses were later determined and list of diagnoses, by location (WHO zone), 2007–2018*

	WHO zone					
Category	AFRO	PAHO	EMRO	EURO	SEARO	WPRO
Undiagnosed disease events, n = 371, no. (%)†	99 (27)	39 (11)	50 (13)	17 (5)	118 (32)	50 (13)
Disease events diagnosed, no./total no. (%)	40/99 (40)	12/39 (31)	15/50 (30)	2/17 (12)	32/118 (27)	26/50 (52)
Main diagnoses	Ebola (n = 5), meningitis (n = 4), yellow fever (n = 4), nodding disease (n = 4)	Leptospirosis (n = 2), Zika virus infection (n = 2)	Cholera (n = 2), dengue fever (n = 2), food poisoning (n = 2), Rift Valley fever (n = 2)	Hantavirus (n = 1), meningitis (n = 1)	Nipah virus infection (n = 6), chikungunya virus infection (n = 3), Crimean Congo hemorrhagic fever (n = 3)	Aflatoxin poisoning (n = 3); hand, foot and mouth disease (n = 3); leptospirosis (n = 3); methanol poisoning (n = 3)

*AFRO, African Regional Office; EMRO, Eastern Mediterranean Regional Office; EURO, Europe Regional Office; PAHO, Pan American Health Organization; SEARO, South-East Asia Regional Office; WHO, World Health Organization; WPRO, Western Pacific Regional Office.
†Two undiagnosed disease events were found in >1 WHO zone.

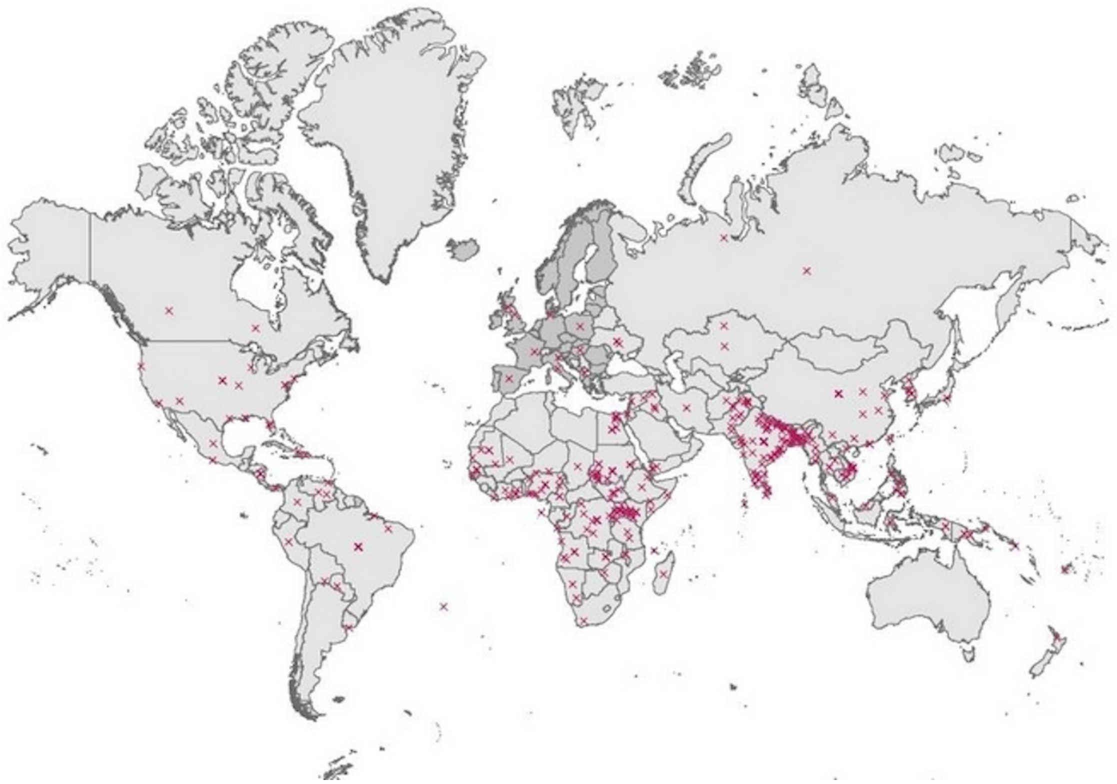

Appendix Figure. Geographic distribution of undiagnosed disease events posted in ProMED-mail from the 1 January 2007 to 14 June 2018 over the world.
